# Differences in Scalp Hair Trace Element Concentrations in Patients with Preserved Left Ventricular Ejection Fraction (HFpEF) Compared with Controls: A Hypothesis-Generating Study

**DOI:** 10.3390/jcm15052029

**Published:** 2026-03-06

**Authors:** Tomasz Urbanowicz, Anetta Hanć, Zofia Kasperowicz, Oliwier Adamczak, Ievgen Spasanenko, Katarzyna Gabriel, Andrzej Tykarski, Zbigniew Krasiński, Beata Krasińska

**Affiliations:** 1 Cardiac Surgery and Transplantology Department, Poznan University of Medical Sciences, 61-848 Poznań, Poland; 2 Department of Trace Analysis, Faculty of Chemistry, Adam Mickiewicz University, 61-614 Poznań, Poland; 3 Cardiology Research Student Group, Poznan University of Medical Sciences, 61-848 Poznań, Poland; 4 Department of Hypertensiology, Angiology, and Internal Medicine, Poznan University of Medical Sciences, 61-848 Poznań, Poland; 5 Department of Vascular, Endovascular Surgery, Angiology and Phlebology, Poznan University of Medical Science, 61-701 Poznań, Poland

**Keywords:** HFpEF, trace elements, hair scalp, magnesium, copper, calcium, lead

## Abstract

**Background:** The pathophysiology of HFpEF is complex and characterized by systemic inflammation, metabolic dysregulation, and endothelial dysfunction. Trace element involvement in redox balance, mitochondrial function, and calcium signaling is postulated. This cross-sectional analysis aimed to investigate possible differences in hair scalp trace element concentrations in patients with HFpEF and controls. **Material and methods**: Fifty-eight consecutive patients were enrolled (HFpEF *n* = 37; controls *n* = 21). HFpEF diagnosis was established using the HFA-PEFF diagnostic algorithm by two independent cardiologists blinded to hair analysis results. Scalp hair samples were analyzed using inductively coupled plasma mass spectrometry (ICP-MS). **Results:** HFpEF patients demonstrated higher hair concentrations of magnesium (17.8 (7.3–47.5) vs. 14.0 (6.7–29.0) µg/g, *p* = 0.037), copper (57.24 (33.87–84.76) vs. 12.96 (9.85–26.02) µg/g, *p* < 0.001), calcium (322 (106–1330) vs. 145 (74–672) µg/g, *p* = 0.006), and lead (0.257 (0.164–0.563) vs. 0.159 (0.079–0.283) µg/g, *p* = 0.03). **Conclusions:** In this exploratory analysis, HFpEF was associated with differences in selected scalp hair trace element concentrations. The interaction between magnesium, calcium, copper, and lead were noted, with higher concentrations in HFpEF phenotypes. These findings are hypothesis-generating and warrant confirmation in larger cohorts incorporating serum/urine measurements and exposure assessment.

## 1. Introduction

Heart failure with preserved ejection fraction (HFpEF) is a prevalent clinical syndrome driven by aging and a high burden of comorbidities such as hypertension, obesity, diabetes, and chronic kidney disease.

It has been reported as the dominant heart failure phenotype [1]. HFpEF is characterized by impaired diastolic relaxation, increased ventricular stiffness, and elevated filling pressures, but accompanied by normal or near-normal left ventricular systolic function [2,3]. This pathology is primarily driven by an aging population characterized by burdens of metabolic disease, including obesity, hypertension, diabetes, and chronic kidney disease [4]. HFpEF requires a deeper mechanistic understanding beyond traditional hemodynamic paradigms to better elucidate its pathophysiology and identify potential mechanisms to improve patient outcomes, as it is associated with high morbidity, recurrent hospitalizations, and limited therapeutic options [5,6]. There was growing evidence suggesting that HFpEF should be considered a systemic disorder driven by low-grade inflammation, endothelial dysfunction, oxidative stress, and metabolic dysregulation rather than an isolated myocardial disease [7,8,9].

The prevailing paradigm emphasizes systemic inflammation, endothelial dysfunction, oxidative stress, and metabolic dysregulation as key contributors to myocardial stiffening and impaired relaxation [10].

Within this framework, the potential impact of trace element homeostasis has emerged as a potentially underexplored contributor to HFpEF pathophysiology. Trace elements play essential roles in mitochondrial energy production, redox balance, calcium handling, immune modulation, and extracellular matrix remodeling, all of which are critically implicated in diastolic dysfunction and myocardial stiffening [11]. Alterations in trace element concentrations, circulating or intracellular, may result in disproportionate effects on cardiovascular structure and function. In HFpEF patients, comorbidity-driven inflammation, combined with age-related multi-organ impairment, may collectively disrupt trace element metabolism, leading to imbalances that further exacerbate myocardial and vascular dysfunction [12]. The role of disturbances in circulating trace element concentrations has been postulated in our previous studies related to atherosclerosis pathology [13,14]. A proper understanding of the bidirectional interactions between HFpEF pathophysiology and trace element concentrations may therefore provide novel insights into disease mechanisms and individualized therapeutic strategies.

The role and correlations of trace elements and toxic elements are significant in the context of cardiovascular disease incidence. Trace elements and toxic metals influence redox homeostasis, mitochondrial function, calcium handling, immune signaling, and extracellular matrix biology. Accordingly, disturbances in long-term elemental status could be associated with HFpEF phenotypes. The ratios of magnesium to calcium and of copper to calcium may be crucial for controlling disease risk, as both magnesium deficiency and excess calcium or copper are associated with increased cardiovascular event risk. Calcium and magnesium regulate key cardiovascular functions, including heart muscle contraction, cellular activity, and expression of heart-related genes. Copper deficiency is associated with heart enlargement and disruptions in calcium metabolism, which may contribute to the development of heart failure. Toxic elements such as lead interfere with calcium binding to calmodulin, disrupting muscle contraction. Lead exposure is also linked to inflammation and oxidative stress, both of which are strongly associated with high blood pressure and vascular diseases. Lead negatively correlates with protective elements such as selenium, promotes pro-inflammatory responses, and damages cardiac cells [15].

Scalp hair provides a stable matrix that can reflect longer-term exposure and incorporation of elements, complementing short-term serum measurements [16].

The pathophysiological backgrounds of HFpEF, including advanced age, female sex, and burden of metabolic disease, including obesity, hypertension, diabetes, and chronic kidney disease, are postulated. The mechanisms underlying disease development and progression are not fully understood and are thought to involve pro-inflammatory activation, leading to endothelial dysfunction, oxidative stress, and metabolic dysregulation. The primary question arises, whether among the possible factors that could modify the HFpEF are trace elements, the co-factors of various enzymatic processes involved in the mentioned mechanisms. From a clinical perspective, trace element dysregulation in HFpEF could be considered a potential therapeutic target.

The aim of this hypothesis-generating study was to compare scalp hair trace element concentrations between patients with HFpEF and a control group without HFpEF. The study of differences in body trace element levels between the analyzed groups was performed to identify possible disturbances that may explain the pathophysiological background of HFpEF and be of interest for future therapeutic approaches.

## 2. Materials and Methods

This prospective, single-center, cross-sectional study enrolled consecutive patients hospitalized in 2024 in the Department of Hypertensiology, Angiology and Internal Medicine. All participants underwent clinical evaluation, laboratory testing, transthoracic echocardiography, and scalp hair trace element analysis. There were 58 consecutive patients screened and included. Based on adjudicated HFA-PEFF scoring, 37 were classified as HFpEF and 21 served as controls.

HFpEF was diagnosed using the Heart Failure Association (HFA)-PEFF diagnostic algorithm. Two cardiologists independently assessed echocardiographic and natriuretic peptide criteria and assigned HFA-PEFF categories; adjudicators were blinded to hair trace element results [17]. Exclusion criteria included LVEF < 50%, at least moderate valvular disease, acute systemic illness or infection, chronic inflammatory/rheumatologic disease, known occupational heavy-metal exposure, mineral supplementation within 3 months, and chemically treated hair (perm or dye). Patients with atrial fibrillation, chronic kidney disease, acute decompensation states, recent mineral supplementation, and hair treatments. Dietary supplements/mineral therapy are considered a contraindication for enrollment.

Any food allergies or intolerances were considered a contraindication for the restrictive diets.

### 2.1. Sample Preparation

The blood samples were taken on admission after at least twelve hours of fasting. Hair samples were collected on admission and prepared according to the standardized protocol.

Hair samples, consisting of scalp hair without chemical treatments such as perm or dye, approximately 2–3 cm in length, were collected from the occipital region near the scalp surface. The collected samples underwent a cleaning procedure comprising sequential washes with prepared detergent solutions, an organic solvent, and deionized water to remove external contaminants, as described in the previously referenced protocol [18]. Subsequently, the hair samples were dried, and finely cut, and approximately 200 mg portions were accurately weighed. The samples were subjected to closed-vessel mineralization using the DigiTube system (SCP Science Ltd., Baie-D’Urfe, QC, Canada) according to the previously described procedure [19]. After mineralization and appropriate dilution, the hair samples were analyzed by ICP-MS (Agilent, Santa Clara, CA, USA) and reported as trace element concentrations per hair weight (ug/g).

### 2.2. Elemental Analysis

The determination of elements was performed using inductively coupled plasma mass spectrometry (ICP-MS 7100x Agilent, Santa Clara, CA, USA). As described in the instrument, it was equipped with an octopole reaction system (ORS) operating in both no-gas and helium modes to reduce spectral interferences. Samples were introduced into the argon plasma (Linde Gas, Cracow, Poland) via a MicroMist concentric nebulizer, quartz Scott double pass spray chamber, and a quartz torch with a quartz injector. Instrumental operating conditions were optimized daily using the Tuning Solution (Agilent, USA). The parameters were set as follows: radiofrequency (RF) power at 1550 W, plasma gas flow rate at 15 L/min, nebulizer gas flow rate at 0.98 L/min, and auxiliary gas flow rate at 0.9 L/min. The ORS mode was employed with helium gas (Linde Gas, Poland) to eliminate spectral interferences. To reduce non-spectral interferences, a 10 μg L^−1^ solution of ^103^Rh and ^159^Tb was used as an internal standard. Calibration curves were constructed over the range of 0.01–100 µg/L for Ag, Al, As, Ba, Cd, Cr, Co, Cu, Li, Mn, Mo, Ni, Pb, Se, Sb, Sn, Sr, V, Ti, U, Zn, and 1–500 µg/L for Ca, Fe, Mg, Na.

### 2.3. Quality Assurance

Certified reference materials, NCS ZC 81002b Human Hair, Beijing, China, and analytical blanks were analyzed to verify accuracy and ensure the traceability of the measurements. Key validation parameters, including linearity, precision, limit of detection (LOD), and trueness, were comprehensively evaluated. The calibration curves were linear, as indicated by the correlation coefficient R, which exceeded 0.9996 for all analytes. The LOD was defined as 3.3 × s/b, where s denotes the standard deviation from 10 blank injections and b represents the slope of the calibration curve. The limits of detection ranged from 0.003 µg/g for Cd to 3 µg/g for Fe. Precision values, calculated as the coefficient of variation (CV, %), ranged from 1.2% to 4.3% for all elements. Trueness was assessed using certified reference material and expressed as recovery percentages, ranging from 93% to 105%.

### 2.4. Statistical Analysis

Continuous variables were reported as medians and interquartile ranges (Q1–Q3) when the data did not follow a normal distribution. Categorical data were presented as numbers and percentages. The Mann–Whitney test was used to compare interval parameters between the analyzed groups. Categorical data were compared using a chi-square test of independence. Statistical analysis was performed using JASP version 0.14.1 (University of Amsterdam, The Netherlands), with a significance level set at *p* < 0.05 (https://jasp-stats.org, accessed 16 December 2020).

### 2.5. Bioethics Committee Approval

The study was conducted in accordance with the Declaration of Helsinki and approved by the Institutional Ethics Committee of Poznan University of Medical Sciences, Poznan, Poland (protocol code 694/20, dated 4 November 2020, for studies involving humans).

## 3. Results

A total of 58 participants were included (HFpEF *n* = 37; controls *n* = 21). In the HFpEF group, 21/37 (57%) were male; in controls, 11/21 (52%) were male. Baseline characteristics are presented in Table 1.

The echocardiographic parameters evaluated in both groups included left ventricular ejection fraction (LVEF) and mass index (LVMI), followed by global longitudinal strain (GLS), left atrial volume index (LAVI), early diastolic mitral annular velocity at the septum (septal E’), diastolic velocity of the lateral wall of the left ventricle (lateral E’), left ventricular filling pressure (E/E’), and relative wall thickness (RWT) (detailed information presented in Table 2).

Key echocardiographic parameters supporting HFpEF classification are summarized in Table 2.

The hair scalp trace element concentrations were compared between both groups (Table 3), indicating significant differences in magnesium (Mg) (17.8 (7.3–47.5) vs. 14.0 (6.7–29.0, *p* = 0.03), copper (Cu) (57.24 (33.87–84.76) vs. 12.96 (9.85–26.02), *p* < 0.001), calcium (322 (106–1330) vs. 145 (74–672), *p* = 0.01), and lead (Pb) (0.213 (0.059–0.700) vs. 0.257 (0.164–0.563), *p* = 0.03).

## 4. Discussion

This cross-sectional study compared scalp hair trace element concentrations between patients with HFpEF and controls. Higher hair concentrations of magnesium, calcium, copper, and lead were observed in the HFpEF group.

The HFpEF epidemiology calls for a thorough investigation regarding the possible underlying mechanism and potential therapeutic targets. The co-morbidities associated with the disease and the echocardiographical criteria have been determined [20,21]. New research for a better understanding of the HFpEF pathophysiology, especially inflammatory-related backgrounds, is required not only to comprehend the possible markers but also to focus on enzymatic co-factors that may be modified in clinical practice. As trace elements are among the best-known modulators of human hemostasis, their potential impact on HFpEF could represent a novel clinical approach. The unique character of our analysis is based not only on the indication that certain trace elements are associated with HFpEF, but also on the identification of possible underlying mechanisms. We strongly believe that the results of our study fill the gap between pathophysiological backgrounds and potential therapeutic targets.

Our primary prospective analysis highlights the potential differences in scalp hair trace element concentrations in the HFpEF profile. Hair element analysis is a method used to assess the body’s mineral status. It provides information about long-term exposure and levels of elements, including trace and toxic elements. Hair reflects intracellular concentrations of these elements, often more reliably than blood tests, making it an essential tool for studying HFpEF pathophysiology. The presented results suggest a mechanistic gap between these enzymatic cofactors (trace elements) and the pathophysiological derangement associated with HFpEF.

The novelty of our study lies in the measurement of trace elements in the body (hair), beyond iron hemostasis. In previous analyses, the high prevalence of iron deficiency was not only associated with worse symptoms but also with reduced quality of life in HFpEF patients [22,23]. This is the first, to our best knowledge, study to present chemical analyses of multiple trace element concentrations. In the previous report [24], the relationships between zinc and copper and heart failure and diastolic dysfunction were investigated.

High magnesium concentration, as a possible contributor to the HFpEF profile observed in our study, has been repeatedly postulated as a predictor of worse outcomes in advanced heart failure [25]. Disturbances in Mg homeostasis may alter mitochondrial respiration, thereby increasing oxidative stress. In the context of molecular mechanisms, it is essential to highlight the potential role of ion homeostasis dysregulation not only in the pathogenesis of HFpEF but also in modulating inflammatory responses and oxidative stress. The aforementioned disturbances in magnesium and calcium balance may contribute to mitochondrial dysfunction, a key factor in the progression of HFpEF. Furthermore, elevated copper levels may exacerbate oxidative stress, leading to endothelial injury and increased extracellular matrix stiffening, as supported by both biochemical and histopathological evidence [26]. Hair is a long-term matrix reflecting element incorporation and exposure over weeks to months, rather than acute circulating concentrations. Accordingly, the observed differences should not be interpreted as serum hypermagnesemia or hypercalcemia.

Calcium, another macronutrient whose higher concentrations were noted in our analysis of HFpEF patients, is recognized as a direct signal for innate immune sensors and can amplify cardiac inflammation relevant to HFpEF. Its involvement in cardiac inflammatory remodeling may increase diastolic tension and induce prohypertrophic and profibrotic signaling, which can both amplify innate immune activation and worsen myocyte diastolic mechanics—a dual contribution to the inflammatory–fibrotic HFpEF phenotype. Both, calcium and magnesium influence excitation–contraction coupling and mitochondrial energetics, and perturbations in these pathways have been implicated in HFpEF.

In our analysis, we noticed higher copper concentration in the HFpEF group. The previous report [24] indicated a correlation between higher Cu levels and left ventricular systolic and diastolic dysfunction. The role of copper dysregulation in the cell cycle, including programmed cell death—known as cuproptosis, has drawn increasing attention to the impact of Cu dyshomeostasis on heart failure risk [27]. Copper participates in mitochondrial enzyme systems and redox signaling; however, mechanistic conclusions from hair levels remain tentative. In a meta-analysis by Liu et al. [28], higher copper concentrations were observed in HF patients than in healthy subjects.

Lead (Pb), unlike the essential metals above, is a purely toxic cation that impairs endothelial nitric oxide synthase function and increases systemic oxidative stress. Lead is a toxic divalent cation largely stored in bone and may be co-released with calcium during states of increased bone turnover. Chronic lead exposure has been linked to vascular stiffness and endothelial dysfunction, but the present study cannot determine exposure source. A previous report [29] suggested that Pb-driven myocardial fibrosis and conduction disturbances could worsen ventricular stiffening and diastolic dysfunction in susceptible hosts, providing a possible mechanistic link between metal exposure and HFpEF phenotypes. Chronic exposure to lead, according to accumulating evidence [30] suggests that exposure to lead may be relevant to the development and progression of HFpEF. Its potential contribution to HFpEF pathophysiology is plausible, related to endothelial dysfunction and myocardial stiffening. Chronic lead exposure not only promotes endothelial injury through oxidative stress but may also directly affect myocardial function [31]. Lead-induced mitochondrial dysfunction is postulated [32,33]. Systemic inflammation represents another critical intersection between lead exposure and HFpEF. Lead stimulates pro-inflammatory cytokine production, including interleukin-6 and tumor necrosis factor-α, and activates nuclear factor κB signaling pathways [34]. Lead exposure may potentiate an inflammatory milieu, thereby promoting pathological changes associated with HFpEF. Fibrotic accumulation within the myocardium increases ventricular stiffness and contributes directly to elevated filling pressures observed in HFpEF.

The possible relationship between the four trace elements presented and HFpEF can be converged on a set of highly conserved biological nodes, such as endothelial dysfunction, mitochondrial redox imbalance, innate immune activation, and altered cardiomyocyte handling. The copper-driven oxidative uncoupling, magnesium-triggered neurohormonal stress, accompanied by lead-induced oxidative stress formation and a calcium-sensing receptor-mediated NLRP3 inflammasome, results in endothelial dysfunction and NO insufficiency that may predispose to HFpEF pathophysiology. Innate immune activation, a potential contributor to HFpEF, may result from Mg^2+^-ATPase dysregulation, copper-induced redox cycling, and lead-induced inhibition of mitochondrial dehydrogenases. The possible interplay among calcium-dependent fibroblast activation, Pb-associated fibrotic transcription, and lipoxygenase hyperactivity, driven by excessive copper, may lead to extracellular matrix stiffening in HFpEF. Disrupted cardiomyocyte function in HFpEF may result from excessive calcium loading, Pb/Ca competition in vascular pathways, magnesium effects on calcium flux, and Cu-induced modifications of calcium-handling proteins.

Potential confounding is important. Medication use (e.g., loop diuretics, mineralocorticoid receptor antagonists), renal function, diet, and unmeasured environmental exposure could influence long-term element status. Therefore, these findings should be considered hypothesis-generating and require replication in larger cohorts incorporating exposure history and serum/urine measurements.

### Study Limitation

This study is limited by its cross-sectional design, modest sample size, and single-center setting. No dietary intake, mineral supplementation history beyond exclusion criteria, or occupational/environmental exposure assessment was available. Serum and urinary trace element measurements were not collected, precluding triangulation with hair concentrations. Multiple comparisons were not performed due to sample size limitation; results should be interpreted as exploratory.

## 5. Conclusions

In this hypothesis-generating cross-sectional study, patients with HFpEF exhibited higher scalp hair concentrations of magnesium, calcium, copper, and lead compared with controls. These associations do not establish causality and warrant confirmation in larger, prospectively phenotyped cohorts with integrated exposure assessment and multimatrix trace element profiling.

## Figures and Tables

**Table 1:** Demographical and clinical characteristics of the analyzed groups.

Parameters	HFpEF Group *n* = 37	Control Group *n* = 21	*p*
Demographics:			
Age (years) (median (Q1–Q3))	71 (65–76)	68 (61–73)	0.46
Sex (males) (*n* (%))	21 (57)	11 (52)	0.76
BMI (kg/m^2^) (*n* (%))	30.3 (25.3–33.1)	27.8 (25.5–32.0)	0.26
Obesity (BMI > 30 kg/m^2^) (*n* (%))	13 (35)	12 (57)	0.11
NYHA status:			
I/II class (*n* (%))	2 (5)	4 (19)	0.18
II class (*n* (%))	28 (76)	16 (76)	0.75
II/III class (*n* (%))	7 (19)	1 (5)	1.00
Clinical characteristics:			
Arterial hypertension (*n* (%))	36 (97)	17 (81)	0.04
Dyslipidemia (*n* (%))	35 (95)	21 (100)	1.00
Diabetes mellitus (*n* (%))	3 (8)	4 (19)	0.23
COPD (*n* (%))	5 (14)	3 (14)	0.95
Stroke (*n* (%))	0 (0)	1 (5)	0.95
PAD (*n* (%))	4 (11)	6 (29)	0.09
Current Smoker (*n*, %)	11 (30)	2 (95)	0.08
Pharmacotherapy			
Beta receptor blockers (*n* (%))	27 (73)	16 (76)	0.38
Angiotensin converting enzyme inhibitor (ACE-I) (*n* (%))	23 (62)	9 (43)	0.83
Angiotensin receptor blocker (ARB) (*n* (%))	5 (14)	5 (24)	0.09
Sodium glucose co-transporter 2 inhibitor (SGLT2i) (*n* (%))	10 (27)	6 (29)	0.19
Loop diuretics (*n* (%))	15 (41)	6 (29)	0.74
Calcium channel blocker (CCB) (*n* (%))	14 (38)	9 (43)	0.72
Mineralocorticoid receptor antagonist (MRA) (*n* (%))	7 (19)	5 (24)	0.27
Statins (*n* (%))	35 (95)	21 (100)	0.96
High statin * dose (*n* (%))	21 (57)	15 (71)	0.60
Laboratory tests			
WBC (×10x9/L) (median (Q1–Q3))	7.23 (6.13–8.51)	6.21 (5.80–8.39)	0.54
Hemoglobin (×10x9/L) (median (Q1–Q3))	8.80 (8.10–9.40)	8.70 (8.30–9.10)	0.71
Hematocrit (×10x9/L) (median (Q1–Q3))	42 (41–44)	42 (39–44)	0.92
Platelets (×10x9/L) (median (Q1–Q3))	234 (186–277)	210 (193–264)	0.58
ALAT (IU/L) (median (Q1–Q3))	26 (21–34)	26 (20–38)	0.75
Glucose (mmol/L) (median (Q1–Q3))	5.60 (5.10–6.00)	5.90 (5.60–6.30)	0.13
HbA1c (%) (median (Q1–Q3))	5.80 (5.60–5.95)	5.80 (5.70–6.00)	0.67
Creatinine (µmol/L) (median (Q1–Q3))	83 (73–96)	82 (69–96)	0.99
Urea (mg/L) (median (Q1–Q3))	345 (305–398)	353 (255–398)	0.75
Total cholesterol (mmol/L) (median (Q1–Q3))	3.93 (3.45–4.33)	4.10 (3.31–5.29)	0.23
HDL (mmol/L) (median (Q1–Q3))	1.49 (1.11–1.66)	1.50 (1.21–1.68)	0.75
LDL (mmol/L) (median (Q1–Q3))	1.93 (1.38–2.47)	1.92 (1.54–3.55)	0.26
Triglycerides (mmol/L) (median (Q1–Q3))	1.05 (0.90–1.41)	1.26 (1.01–1.64)	0.14
Lipoprotein a (mg/mL) (median (Q1–Q3))	10.00 (2.40–23.85)	7.90 (4.20–14.90)	0.49
CK-MB (ng/mL) (median (Q1–Q3))	2.105 (1.337–2.910)	1.520 (0.705–2.055)	0.11
BNP (pg/mL) (median (Q1–Q3))	325 (256–502)	102 (74–148)	<0.001

Abbreviations: ALAT—alanine aminotransferase, BMI—body mass index, BNP—brain natriuretic peptide, CK-MB—creatinine kinase MB, COPD—chronic obstructive pulmonary disease, Hb1Ac—glycemic hemoglobin, HDL—high density lipoprotein, L—liter, kg—kilogram, LDL—low-density lipoprotein, m^2^—square meter, mL—milliliter, ng—nanograms, mmol—millimole, *n*—number, NYHA—New York Heart Association, PAD—peripheral artery disease, pg—picogram, µmol—micromole, WBC—white blood count, %—percentage. * daily dose of at least 40 mg of atorvastatin or 20 mg of rosuvastatin.

**Table 2:** Echocardiographic characteristics of the analyzed groups.

Parameters	HFpEF Group *n* = 37	Control Group *n* = 21	*p*
HFA-PEFF score echocardiographic elements:			
Septal E’ (cm/s) (median (Q1–Q3))	5 (4–7)	8 (7–10)	0.003
Lateral E’ (cm/s) (median (Q1–Q3))	8 (6–8)	9 (7–11)	0.02
E/E’ (median (Q1–Q3))	19.2 (17.8–21.2)	7.8 (6.9–9.2)	0.06
E/E’ > 14 (*n* (%))	37 (100)	0 (0)	0.01
GLS (%) (median (Q1–Q3))	15 (13–19)	19 (16–21)	0.02
GLS < 16% (*n* (%))	30 (81)	1 (5)	0.02
LAVI (mL/m^2^) (median (Q1–Q3))	29 (24–34)	24 (19–26)	0.002
LAVI > 34 mL/m^2^ (*n* (%))	20 (54)	0 (0)	<0.001
LAVI 29–34 mL/m^2^ (*n* (%))	17 (46)	0 (0)	<0.001
LMVI (median (Q1–Q3))	106 (85–125)	83 (68–92)	0.001
RWT (median (Q1–Q3))	0.46 (0.41–0.56)	0.37 (0.35–0.41)	<0.001
HFpEF diagnosis based on HFA-PEFF score: Unlikely (*n* (%)) Intermediate (*n* (%)) Probable (*n* (%))	0 (0) 13 (35) 24 (65)	21 (100) 0 (0) 0 (0)	<0.001
Echocardiographic parameters			
LVED (mm) (median (Q1–Q3))	49 (43–52)	43 (42–48)	0.02
LVESd (mm) (median (Q1–Q3))	35 (32–41)	36 (26–38)	0.26
LVEDVi (mm/) (median (Q1–Q3))	58.6 (48.9–67.4)	47.4 (40.6–58.6)	0.01
LVESV (mm^3^) (median (Q1–Q3))	29 (23–35)	27 (21–31)	0.10
LVEF (%) (median (Q1–Q3))	54 (51–60)	54 (51–58)	0.87
E/A > 0.8 (*n* (%))	0.62 (0.56–0.74) 37	0.98 (0.85–1.19)	<0.001
E/A > 0.8 (median (Q1–Q3))	37 (100)	0 (0)	<0.001
GWI (median (Q1–Q3))	1766 (1490–1977)	1602 (1231–2021)	0.47
GCW (median (Q1–Q3))	1984 (1657–2126)	1928 (1438–2310)	0.88
GWW (median (Q1–Q3))	137 (93–256)	192 (107–296)	0.38
GWE (median (Q1–Q3))	93 (86–96)	90 (83–94)	0.33

Abbreviations: cm—centimeter, E/E’—left ventricular filling pressure, GLS—global longitudinal strain, GWE—global work efficiency, GWI—global work index, GWW—global wasted work, HFA-PEFF—Heart Failure Association: Pretest assessment, echocardiography and natriuretic peptide, functional testing and final etiology, HFpEF—heart failure with preserved ejection fraction, Lateral E’—diastolic velocity of the lateral wall of the left ventricle, LAVI—left atrial volume index, LVMI—left ventricular mass index, mL—milliliter, mm^3^—cubic millimeter, m^2^—square meter, *n*—number, RWT—relative wall thickness, s—second, septal E’—early diastolic mitral annular velocity at the septum, Q—quartile.

**Table 3:** Hair scalp trace elements analysis.

Trace Elements (Median (Q1–Q3)) [µg/g Hair]	HFpEF Group *n* = 37	Control Group *n* = 21	*p* **
Li (Lithium)	0.016 (0.007–0.021)	0.017 (0.008–0.024)	0.13
Na (Sodium)	141 (100–179)	135 (111–171)	0.31
Mg (Magnesium)	17.8 (7.3–47.5)	14.0 (6.7–29.0)	0.037 *
Al (Aluminum)	5.32 (2.96–10.41)	3.50 (2.24–5.33)	0.72
Ca (Calcium)	322 (106–1330)	145 (74–672)	0.006 *
Ti (Titanium)	0.406 (0.244–0.555)	0.371 (0.302–0.462)	0.64
V (Vanadium)	0.038 (0.026–0.057)	0.038 (0.030–0.061)	0.25
Cr (Chromium)	0.967 (0.669–1.291)	0.900 (0.753–1.303)	0.17
Mn (Manganese)	0.182 (0.128–0.398)	0.179 (0.099–0.299)	0.39
Fe (iron)	7.70 (5.15–15.54)	7.17 (5.78–11.17)	0.75
Co (Cobalt)	0.017 (0.011–0.025)	0.013 (0.011–0.025)	0.85
Ni (Nickel)	0.950 (0.715–1.149)	0.866 (0.616–1.202)	0.95
Cu (Copper)	57.24 (33.87–84.76)	12.96 (9.85–26.02)	<0.001
Zn (Zinc)	137 (81–162)	146 (68–207)	0.76
As (Arsenic)	0.027 (0.023–0.058)	0.038 (0.027–0.078)	0.15
Se (Selenium)	0.371 (0.265–0.613)	0.424 (0.207–0.800)	0.60
Sr (Strontium)	1.068 (0.305–3.493)	0.354 (0.134–1.235)	0.05
Mo (Molybdenum)	0.133 (0.110–0.173)	0.145 (0.121–0.203)	0.25
Ag (Silver)	0.065 (0.036–0.152)	0.048 (0.028–0.208)	0.29
Cd (Cadmium)	0.012 (0.008–0.026)	0.009 (0.007–0.019)	0.36
Sn (Tin)	0.103 (0.055–0.359)	0.092 (0.054–0.201)	0.45
Sb (Antimony)	0.012 (0.007–0.021)	0.017 (0.009–0.037)	0.14
Pb (Lead)	0.257 (0.164–0.563)	0.159 (0.079–0.283)	0.03 *
U (Uranium)	0.003 (0.002–0.016)	0.003 (0.002–0.006)	0.35

Abbreviations: HFpEF—heart failure with preserved ejection fraction, *n*—number, Q—quartile, *—statistically significant., ** *p* values unadjusted for multiple comparisons; exploratory/hypothesis-generating.

## Data Availability

The data supporting the reported results are available upon reasonable request by contacting the corresponding author. The restriction relies on privacy or ethical reasons.
